# How to Decrease the Viscosity of Suspension with the Second Fluid and Nanoparticles?

**DOI:** 10.1038/srep03137

**Published:** 2013-11-05

**Authors:** Menghan Xu, Haifeng Liu, Hui Zhao, Weifeng Li

**Affiliations:** 1 Key Laboratory of Coal Gasification and Energy Chemical Engineering of Ministry of Education, East China University of Science and Technology, P.O. Box 272, No.130 Meilong Road, Shanghai 200237, People's Republic of China

## Abstract

According to recent research reports, addition of small amounts of a secondary fluid to a suspension could dramatically increase viscosity of suspension. Results of this study indicate another interesting behavior that the secondary fluid could form a thin hydrophobic membrane around particle surface and significantly decrease the viscosity and yield stress of the suspension. To enhance the surface hydrophobicity, hydrophobic nanoparticles (nano-CaCO_3_) were added to the hydrophobic membrane of particles to improve the surface roughness, and to generate composite particles having a hierarchical structure similar to the micromorphology of lotus leaf. This composite particle has a higher contact angle, and the suspension of composite particles has a lower viscosity and a lower yield stress.

Suspensions of particles are ubiquitous in nature and are important in both of scientific and industrial fields such as food, plastics, pharmaceutical and coal gasification. Much effort has been made in controlling the flow behavior and rheological properties of suspension. It was found that the rheological behavior was usually controlled by the particle volume fraction, the particle shape, repulsive electrostatic force between particles, the spatial arrangement, and nature of the bulk fluid^1^,^2^,^3^,^4^,^5^. Physicochemical properties of particle surface, such as the functional groups, roughness, wettability and the effect of surfactants, may also influence the rheological properties^6^,^7^,^8^. In practical application, to produce and deliver a suspension with a low volume ratio of particles to suspension and with high viscosity is often required, such as the dye-pigment. However, it also needs the suspension with low viscosity and high volume ratio of particles to suspension, such as the coal-water slurry (CWS).

The addition of a small amount of fluid to a suspension may dramatically change the macroscopic properties of the material, and attractive capillary forces between two grains deforms the grains elastically, yielding a “spring constant” for further deformation^9^. Along the same lines, just a bit of water can enable one to turn a pile of dry sand into a spectacular sandcastle^10^. Koos and Willenbacher found that adding small amount of a secondary fluid, immiscible with the primary fluid in suspension, could create pendular bridges or capillary forces between particles, and dramatically altering the bulk rheological behavior from predominantly viscous or weakly elastic to highly elastic or gel-like^11^,^12^,^13^,^14^. When the secondary fluid preferentially wets the particles, it forms a pendular meniscus around the contact point between particles. Once such a meniscus was formed, the interfacial tension would draw the particles together and formed an elastic network, it is called pendular state^12^. If the second immiscible fluid did not preferentially wet the particles, it would agglomerate the particles and create sample-spanning network structures due to the strong capillary forces from the bulk wetting fluid. In contrast to the pendular state, this is called capillary state^11^,^13^.

The two-fluid suspension is complex and behaves in a variety of ways. A question may arise whether the secondary fluid increase the viscosity of suspensions. The lotus leaf has hydrophobic nature, which has a thin botanical wax film present on the leaf surface. This unique micronano hierarchical structure was constructed by the sparsely populated micropapillae with nanosized wax crystals to make it a superhydrophobic surface^15^,^16^,^17^. These result in Cassie-Baxter state and a high contact angle between water droplet and the leaf surface^18^,^19^. To mimic these biostructures, the second immiscible fluid works as an adhesive coating the particle surface and forming a thin hydrophobic membrane similar to botanical wax film present on the leaf surface. Hydrophobic nanoparticles could achieve stable adhesion on the hydrophobic membranes due to the strong attraction. By adding these two materials to particles, it is possible to create a “composite particle” with microbumps and nanobumps similar to those on lotus leaf, and make the surface of particles more hydrophobic and rough like a superhydrophobic surface. As a result, during the preparation of suspension, the particle's hydration layer^20^,^21^ may become thinner, leading to repulsion of water from the surface of particles. As a consequence, the amount of free water may be increased and the viscosity of suspension would be reduced dramatically. In order to prevent the secondary fluid from aggregation of particles by forming pendular bridges, a small amount of dispersant either a non-surface active polymer or a surface-active substance was added to suspension to improve the separation of particles and also to prevent them from settling or clumping^22^,^23^. Such dispersant can be used to prepare suspension of composite particles. This approach is very convenient to reduce the viscosity and improve the liquidity of suspensions. The notable feature of the preparation is its operation without special environment and intricate instruments.

In this paper, a lotus leaf like structure has been created using hollow glass bead, polyvinyl chloride (PVC) and Brown coal, combined with kerosene and nanometer calcium carbonate (nano-CaCO_3_). The nano-CaCO_3_ suspended in kerosene is referred to as kerosene suspension of nano-CaCO_3_ (KSNC). The rheological behaviors and the microscopic structure of suspensions have been investigated to explain the mechanisms in which the secondary fluid and nanoparticles influence the rheology of suspensions. The microbumps and nanobumps were also studied by analyzing the contact angles between water and the composite particles.

The model of the composite particles is shown in Figure 1. The secondary fluid, kerosene as was thoroughly mixed with nano-CaCO_3_ to generate another secondary fluid—KSNC. Then KSNC was added to the particles. It spontaneously coated the outer surface of particles and forms hydrophobic membranes. By virtue of its strong hydrophobic property, the nano-CaCO_3_ would locate on the surface of hydrophobic membranes. The kerosene and nano-CaCO_3_ served as the first and secondary structure to form a hierarchical structure similar to the micromorphology of lotus leaf. Using this method, hydrophobic and rough “particle/kerosene + nano-CaCO_3_” composite particles with microbumps and nanobumps on the surface were prepared.

## Results

### Viscosity and yield stress of suspension with the different secondary fluid

**Figure 2:**
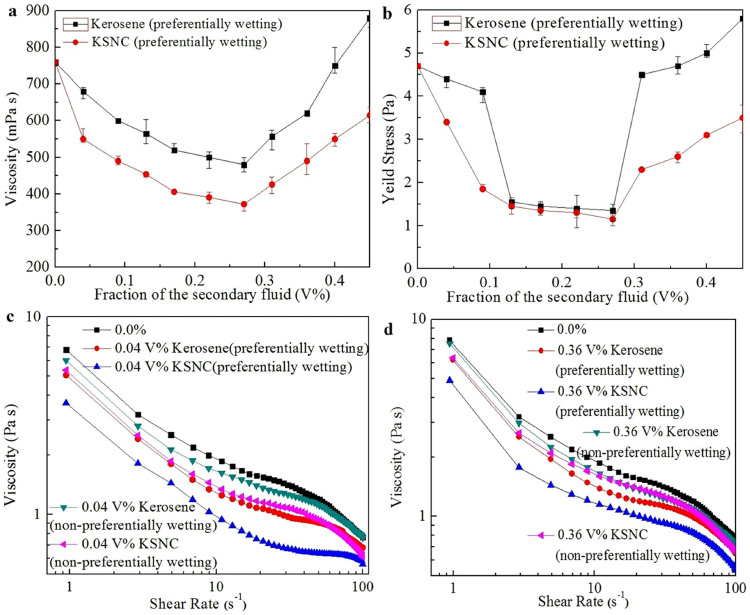
Effect of the addition of secondary fluid on the rheology of suspension prepared using hollow glass beads. Viscosity (a) and yield stress (b) at a shear rate of 100 s^−1^, for varying volume percentages of the secondary fluid (kerosene and KSNC) that preferentially wets particles; (c) Flow curves of 0.04 V% (0.1 wt%) of secondary fluid (kerosene and KSNC) preferentially and non-preferentially wets particles. (d) Flow curves of 0.36 V% (0.8 wt%) of secondary fluid (kerosene and KSNC) preferentially and non-preferentially wets particles. Error bars in (a) and (b) indicate repeatability error.

Hollow glass beads in water were used as a model system to evaluate the performance of the secondary fluid (kerosene and KSNC) in suspensions. The amount of secondary fluid is varied between 0.0 and 0.45 V% (1.0 wt%, on a dry solid basis). The solids loading of all the suspensions is 56.7 V% (32.0 wt%) with 1.0 wt% of dispersant (on the basis of the weight of the dry solid) of sodium naphthalene sulfonate formaldehyde condensate (MF). Each suspension was characterized by measuring steady-state viscosity and yield stress, as shown in Figure 2a and Figure 2b, respectively. The yield stress of suspension was measured according to the procedure described by Møller P.C.F et al^24^,^25^,^26^. For stresses smaller than that of the critical stress, the viscosity becomes so high that buildup of structure is prevented from destruction. On the other hand, for a stress slightly higher than that of the critical stress, destruction of the microstructure occurs, causing viscosity to gradually decrease and reach a low steady-state value. The measurements of yield stress are shown in Supplementary Figures S2.

It is obvious that the viscosity of suspension is very sensitive to the addition of secondary fluid which preferentially wetted the particles. As shown in Figure 2a, the viscosity of the suspension evaluated at a single shear rate of 100 s^−1^ decreased sharply as dosage of kerosene was increased from 0.0 to 0.27 V% (0.6 wt%), and the effect of KSNC is much higher than that of the kerosene without nano-CaCO_3_. It can also be seen that 0.04 V% (0.1 wt%) dosage of KSNC with 0.13 V% (0.3 wt%) of pure kerosene has the same effect. As with the viscosity, the yield stress of suspensions with 0.04 V% dosage of KSNC (Figure 2b) decreased by a factor of four compared to the single-fluid suspension. The lowest yield stress and viscosity occurred at a dosage of 0.27 V% of secondary fluid. However, for all suspensions containing kerosene or KSNC, the viscosities and yield stresses increased for dosages exceeding 0.27 V%. The unmodified particle suspension has a larger yield stress, because the particles are hydrophilic and that would connect with water easily. However, after preferentially wetting the surface, the links between water and particles deteriorated. Thus, the absorption of dispersants on the modified particles increases accordingly.

### The influence of ways of adding secondary fluid on rheology

The ways of adding the secondary fluid may make great influence on the rheology of suspensions. In addition to the secondary fluid preferentially wetting the particles, a second immiscible fluid was added to the water suspension and uniformly mixed. Flow curves of each mixture with 0.04 V% (0.1 wt%) and 0.36 V% (0.8 wt%) of the second fluid are shown in Figure 2c, Supplementary Figure S2a, Figure 2d, and Supplementary Figure S2b. The viscosity and yield stress of suspensions without preferentially wetting the particles with second fluid were much higher than those of suspensions in which particles were preferentially wetted. The reason for this behavior is that the secondary, non-preferentially wetting fluid does not link to the corresponding functional groups on the particle surface to create hydrophobic membranes. Therefore there was no increase in the hydrophobicity of particles. As a result, the second fluid has insignificant effect on the viscosity and the yield stress values of suspensions. The results show that the viscosity of suspensions in which the second fluid did not preferentially wet the particles is lower than that of the single-fluid suspension. As a part of the dispersant was absorbed on the secondary fluid, it does not agglomerate the particles but experience a repulsive interaction. In addition, the secondary fluid increases the effective volume fraction of the suspensions slightly, and therefore, the viscosity of the suspensions slightly decreases.

### Forms of existence of secondary fluid in suspension

**Figure 3:**
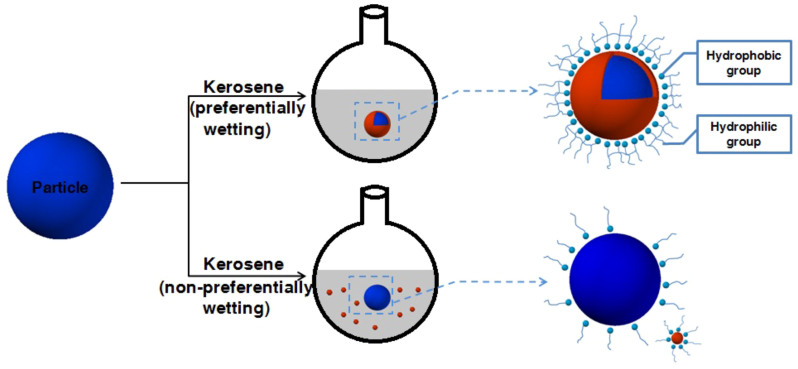
Schematic drawing of particle suspended in water with kerosene wetting the particles both preferentially and non-preferentially and the dispersion mechanism of dispersant in suspension.

The way in which secondary fluid exists in suspensions is shown in Figure 3. It shows that if kerosene as the secondary fluid preferentially wetted the particles, it would form hydrophobic membranes and remained stable in water suspensions. On the other hand, if kerosene was added to the water suspension in the final step, instead of covering the surface of particles, it would form small droplets of varying size in whole suspensions. The hydrophobic groups of a dispersant when presented in a suspension may easily adsorb on the surface of kerosene membranes or oil droplets, and the polyether inside chains of dispersant molecules would form stable adsorbed layers. When the polymer chains were completely dissolved and appropriately unfolded in water, their steric hindrance would make the particles to disperse and more stability. Unless the secondary fluid either preferentially or non-preferentially wetted the particles, it would not able to form a pendular meniscus around the contact point between particles or agglomerate them and created sample-spanning network structures. This also explains that the dispersant more easily wets surface of the composite particles than non-composite ones.

**Figure 4:**
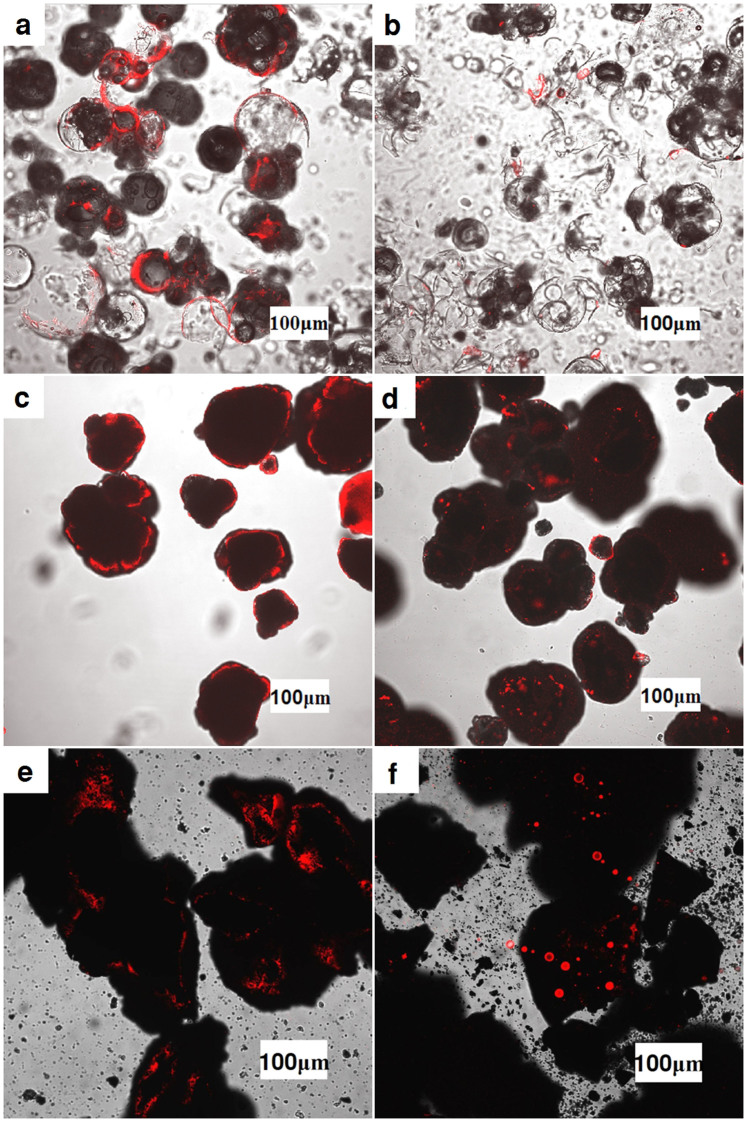
Composite images of suspensions with the second fluid in different adding methods. Hollow glass beads in water with (a) 0.04 V% of preferentially wetting kerosene and with (b) 0.04 V% of non-preferentially wetting kerosene. PVC in water with (c) 0.04 V% of preferentially wetting kerosene and with (d) 0.04 V% of non-preferentially wetting kerosene. Brown coal in water with (e) 0.04 V% of preferentially wetting kerosene and with (f) 0.04 V% of non-preferentially wetting kerosene. All these composite images are composed of the hydrophobic fluorescent dye images in red, used to highlight the kerosene, merged onto the corresponding unfiltered real-light images (see Supplementary Figure S5).

The laser scanning confocal microscope was used to observe the morphology of particles in suspensions. Images of the suspensions with three different particles in water with kerosene wetting the particles both preferentially and non-preferentially were obtained, as shown in Figure 4. Hydrophobic fluorescent dye [DiIC1(5) iodide, Fanbo Biochemicals Co. Ltd.] was used to indicate the location of kerosene. The state of kerosene preferentially wetting the particles is shown in Figures 4a, 4c and 4e. The red circular outlines are clearly visible, indicating that the second fluid is attached to the surface of particles to form hydrophobic membranes, and that pendular bridges are not formed. The other state of kerosene not preferentially wetting the particles was obtained by directly adding it to the suspensions. The corresponding images of different particle suspensions obtained are shown in Figures 4b, 4d and 4f. It can be seen that kerosene is dispersed in the suspension as globular drops. So the addition of secondary fluid does not modify the particle's surface, and it has insignificant effect on rheology of the suspension.

### Effect of secondary fluid and nanoparticles on contact angle

**Figure 5:**
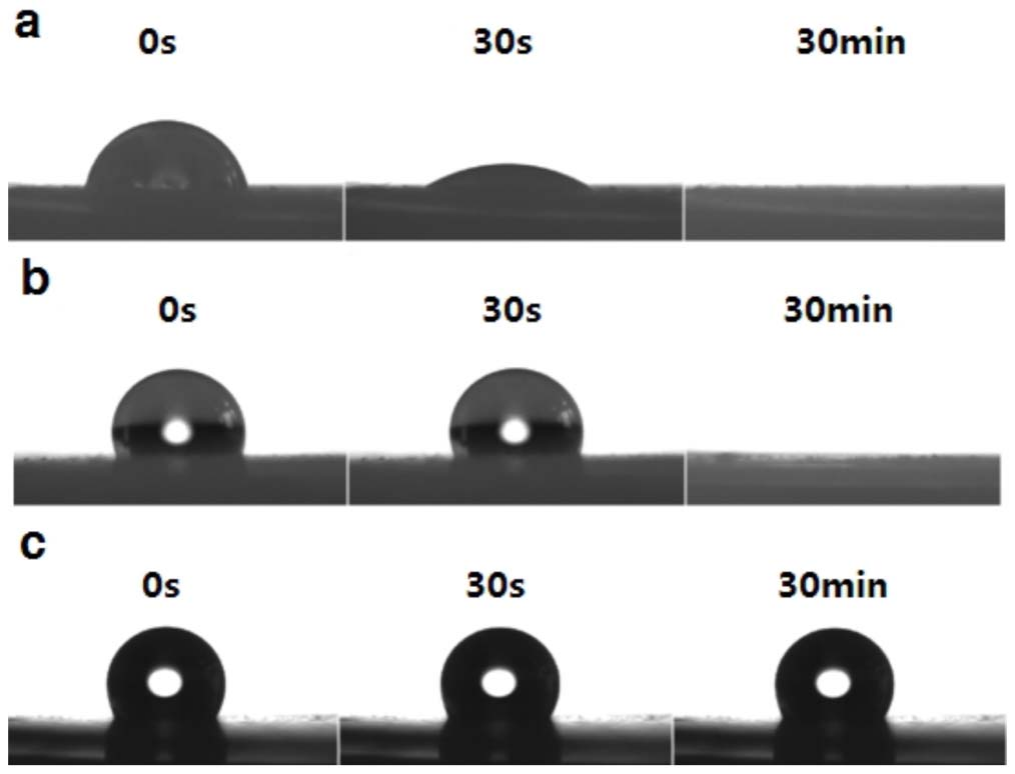
Images of a water droplet sitting on the slices of PVC particles in 0 s, 30 s and 30 min. (a), PVC particles without addition of secondary fluid. (b), PVC particles with 0.09 V% of kerosene. c, PVC particles with 0.09 V% of KSNC.

To study the effect of nanoparticles on rheology of suspensions, the contact angle of PVC particles was measured to verify the changes occurred in hydrophobicity of particles before and after modification. The particles without addition of the secondary fluid gave a contact angle of 80.7 ± 1.0° for water (Figure 5a). The water droplets sank through the slice surface in 30 s. For the particles modified by kerosene, a higher contact angle of 110.7 ± 2.0° for water was obtained. The water is able to maintain in droplet state for 5 min on the slice surface. For the composite particles with nano-CaCO_3_, a contact angle of 127.1 ± 2.0° for water was measured. In this case, water maintained the droplet state for 10 h. These results show that the hierarchical structure has great effect on the wettability, and that nanoparticles cause formation of microbumps and nanobumps on the surface improving hydrophobicity and roughness of the particles.

## Discussion

In this study, the effects of the second immiscible fluid on rheology of particle suspensions containing a dispersant are investigated. The addition of secondary fluid which preferentially wetted particles dramatically changes the rheological properties of the mixture. The secondary fluid improves the hydrophobicity of the particles, leading to increase in free water in suspension and significant decrease in the viscosity and yield stress. The micronano hierarchical structure similar to lotus leaf is developed by adding hydrophobic nanoparticles, and a highly water repellent microparticle surface is fabricated under normal conditions. The results are in agreement with the theory which explains that addition of hydrophobic nanoparticles decreases the yield stress and viscosity of a suspension, and increases contact angles for water.

This method can be widely used in industrial applications because of its convenience and low cost, and it is applicable to a wide variety of particles of different size, shape and surface properties due to the nonselective adhesion of the secondary fluid. Significantly, this method can be used in preparation of highly concentrated coal-water slurry in gasification, the small, solid particles of coal and a dispersant dispersed in water. The surface modification technology of granules will lay a path to design of new materials that will find application in food and materials processing, cement production, and biological field.

## Methods

### Materials

The particles used in this research are hollow glass beads (Shanghai Huijingya nano-materials Co., Ltd., China), PVC and Brown coal from Yunnan, as summarized in Supplementary Table S1 and in Supplementary Figure S1. Industrial kerosene, MF, nano-CaCO_3_ (For nano-CaCO_3_, the contact angle for water is θ = 110°) and deionized water were also used.

Hollow glass beads and PVC particles were dried in an oven at 105°C for 24 h. These two samples were then screened through 200–325 mesh screens and 80–120 mesh screens to obtain the desired size distribution, respectively. Brown coal was dried in an oven at 105°C for 24 h, and comminuted in a ball mill to produce two optimum particle size distributions. The mean volume diameter of each type of particle is reported in Supplementary Table S1. For the preparation of CWS, coarse particles and fine particles were mixed in a mass ratio of 6:4.

### Sample preparation

Nano-CaCO_3_ particles of 50 nm in diameter were added to kerosene (nano-CaCO_3_ and kerosene were in the proportion of 1:5 wt:wt) and were mixed thoroughly to generate KSNC. The secondary fluid (kerosene or KSNC) was added slowly to the dried particles in a stainless steel vessel. The mixture was continuously stirred at a constant speed of 500 r/min for 30 min to ensure the homogenization of the secondary fluid. The particles with the secondary fluid are called the composite particles. First, it is ensured that nano-CaCO_3_ particles are adhered to the coal particle's surface. For achieving this, part of kerosene is added to coal particles to make the particle's surface completely hydrophobic, and then rest of KSNC is added as mentioned above.

To prepare uniform suspension, the test particles were mixed with the required quantity of bulk fluid (deionized water) containing 1.0 wt% dispersant MF (on the basis of the weight of the dry solid) at a constant speed of 1000 r/min for 20 min. About 200 ml of suspension was allowed to stand for 5 min to release the entrained air before taking measurements.

### Particle size measurements

The size of the particle was determined by automatic laser granularity analyzer (Malvern mastersizer 2000) by suspending in ethanol and subjecting to ultrasonic diffusion. The mean volume particle diameters of the test samples are shown in Supplementary Table S1.

### Rheology measurements

Rheological property measurements were carried out using rotating-type rheometer (model Malvern Bohlin CVO). The rheometer consists of a cup centered on a turntable with a rotor concentrically suspended within it. The sample was placed in the annular space between the inner rotor and outer cylinder in measurement. The temperature was held constant at 25°C. For measurement of viscosity, the shear rate was varied smoothly from 0 to 100 s^−1^ in 100 s. Later on, the shear rate was kept constant at 100 s^−1^ for 30 s for obtaining additional viscosity measurements. The results are averaged for determining the viscosity. The shear rate was then gradually decreased from 100 to 0 s^−1^ in 100 s.

The determination of yield stress: It is the stress at which the suspension begins to flow, such as the point at which slope of the strain and shear stress curve (stain as a function of the shear stress) changes from a very low to a high value. Also at yield stress a rapid reduction occurred in the measured viscosity. The shear stress ramp was conducted between 0 Pa and 5 Pa (or 10 Pa) with 50 sample points over 100 s. Experiments were repeated three times to ensure that the ramp limits are sufficient and results are consistent.

### Microstructure of particles and suspension

The microstructure of particle was studied using scanning electron microscope (HITACH SU1510). The microstructure of suspension was observed using laser scanning confocal microscope (Nikon A1R). The composite images (Figure 4 of the main text) were created by merging an unfiltered real-light image with a filtered, UV-light image using fluorescent dye stained in the kerosene, as shown in Supplementary Figure S5. The intensity of the UV-light image was marked red in the composite image for clarity.

### Contact angle measurements

Contact angles were measured by means of static drop method using an optical tensionmeter (Theta Lite). First, the test particles were mixed with a certain dosage of kerosene (or KSNC) to produce the composite particles. Then, the particles were pressed at a pressure of 12.5 MPa to form slices with a diameter of 13 mm and thickness of 1 mm. The slices were stored in a closed container to prevent evaporation of kerosene during the measurement. The contact angles were measured in air. Three slices were made for each sample and three measurements were obtained at different places on each slice. Thus, reported values are averages of nine measurements for one sample. Measurements were obtained to determine how long water droplets remained on the slices. For these measurements, the slices were stored in a closed container to prevent evaporation of the water droplets and kerosene.

## Author Contributions

Conceived and designed experiments: H.L., M.X. Performed the experiments and analyzed the data: M.X., H.Z. Theoretical calculation: W.L., H.Z. Wrote the paper: M.X., H.L., H.Z., W.L. All authors contributed to the interpretation and discussion the manuscript.

## Supplementary Material

Supplementary Information Supplementary information

## Figures and Tables

**Figure 1:**
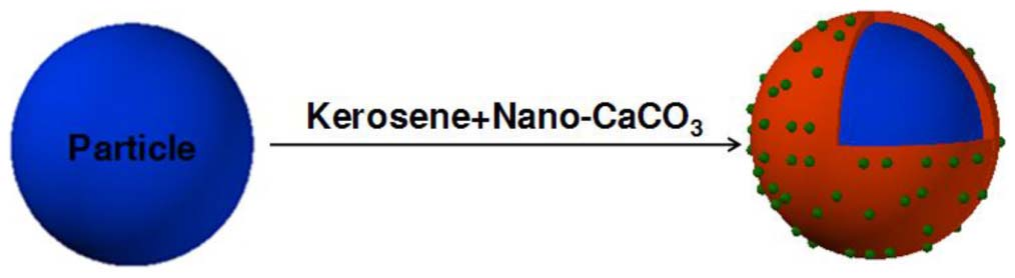
Schematic drawing of the “hollow glass bead/kerosene + nano-CaCO_3_” composite particle.
