# Supplementary material for: How to Decrease the Viscosity of Suspension with the Second Fluid and Nanoparticles?
Source: Sci Rep. 2013 Nov 5;3:3137. doi: 10.1038/srep03137 (PMC3817430; doi:10.1038/srep03137)
Supplement: Supplementary Information [file srep03137-s1.pdf]

How to Decrease the Viscosity of Suspension with the Second Fluid and Nanoparticles?

Menghan Xu, Haifeng Liu*, Hui Zhao, Weifeng Li

Supplementary Information:

The particles used in this research are summarized in Table S1 and shown in Fig. S1.

For hollow glass bead, the relationship between yield stress of suspension and secondary fluid dosage is depicted in Fig. S2.

For PVC and Brown coal, the amount of secondary fluid is varied between 0.0 and 0.76 V% (or 1.69 V%, on a dry solid basis). The solid content of PVC and Brown coal suspensions were 51.0 V% (56.0 wt%) and 46.5 V% (54.0 wt%) respectively. The relationships between rheology of suspension and the secondary fluid dosages are depicted in Fig. S3 and Fig. S4.

The composite images (Fig. 4 of the main text) were created by merging an unfiltered, real-light image with the filtered, UV-light image used fluorescent dye [DiIC1(5) iodide, Fanbo Biochemicals Co. Ltd.] dyed in the kerosene, as shown in Fig. S5. The intensity of the UV-light image was colored red in the composite image for clarity.

The experimental results on Brown coal particles are shown in Fig. S6. The particles without addition of secondary fluid gave a contact angle of 52.6 ± 1.0° for water (see in Fig. S6A). The water droplets sank through the slice surface in 30 seconds. For the particles modified by kerosene, a higher contact angle of 117.8 ± 2.0° for water is obtained. The water is able to maintain in droplet state for 6 minutes on the slice surface. For the composite particles with nano-$CaCO_3$, a contact angle of 129.2 ± 2.0° for water is measured. In this case, water maintained the droplet state for 10 h.

Supplementary Table S1│ The mean volume diameter [D[4,3]] of the test particle samples.

Solid	*Diameter[4,3] (μm)*
Hollow glass bead	44
PVC	132
Brown coal	34<br>141

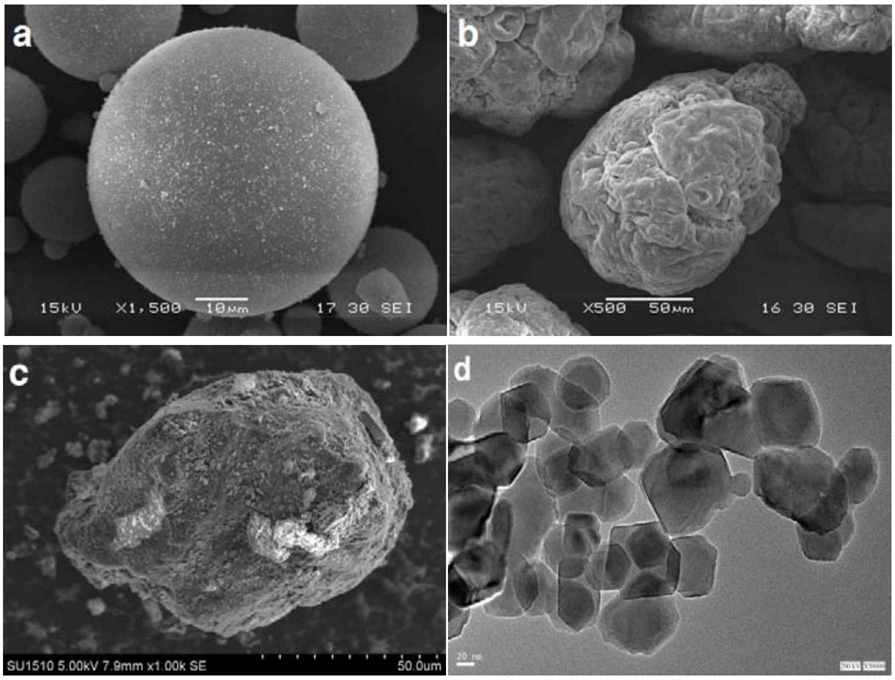

Supplementary Figure S1│ Microscopic images of the particles used in this study. a, SEM image of hollow glass bead; **b**, SEM image of PVC; **c**, SEM image of Brown coal; **d**, TEM image of nano-$CaCO_3$.

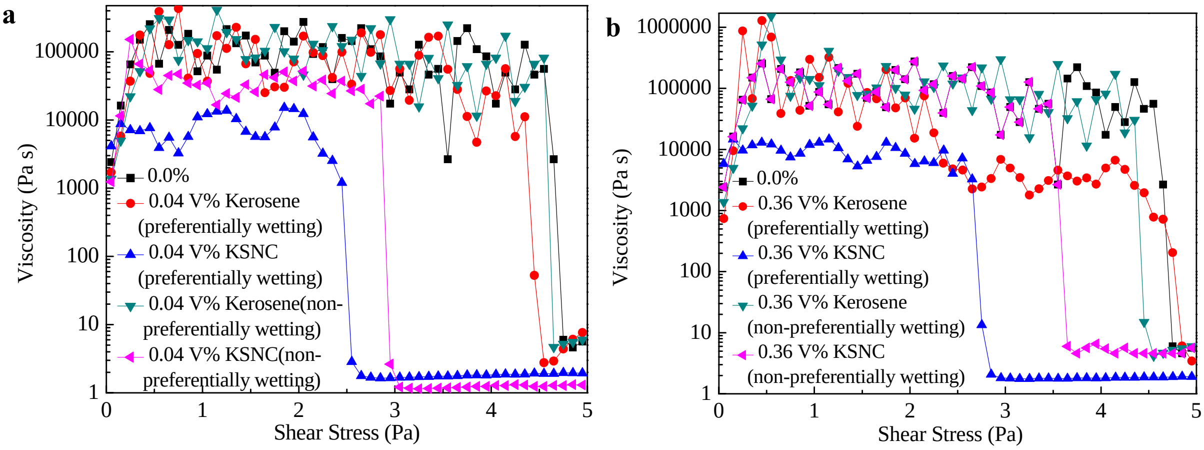

Supplementary Figure S2 | Effect of the addition of secondary fluid on the yield stress of suspension prepared using hollow glass beads. (**a**) Flow curves of 0.04 V% of secondary fluid (kerosene and KSNC) preferentially and non-preferentially wets particles. (**b**) Flow curves of 0.36 V% of secondary fluid (kerosene and KSNC) preferentially and non-preferentially wets particles.

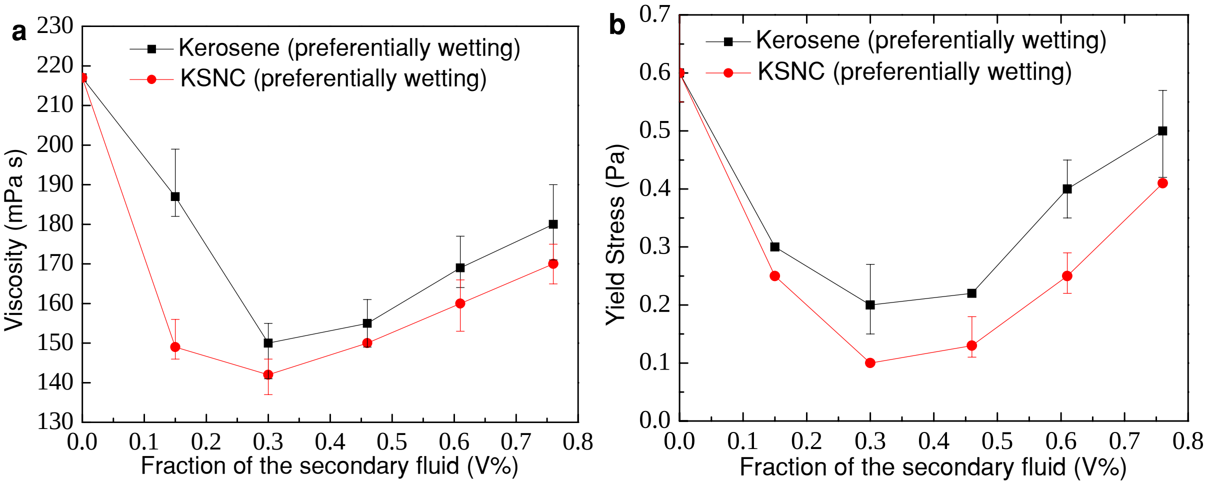

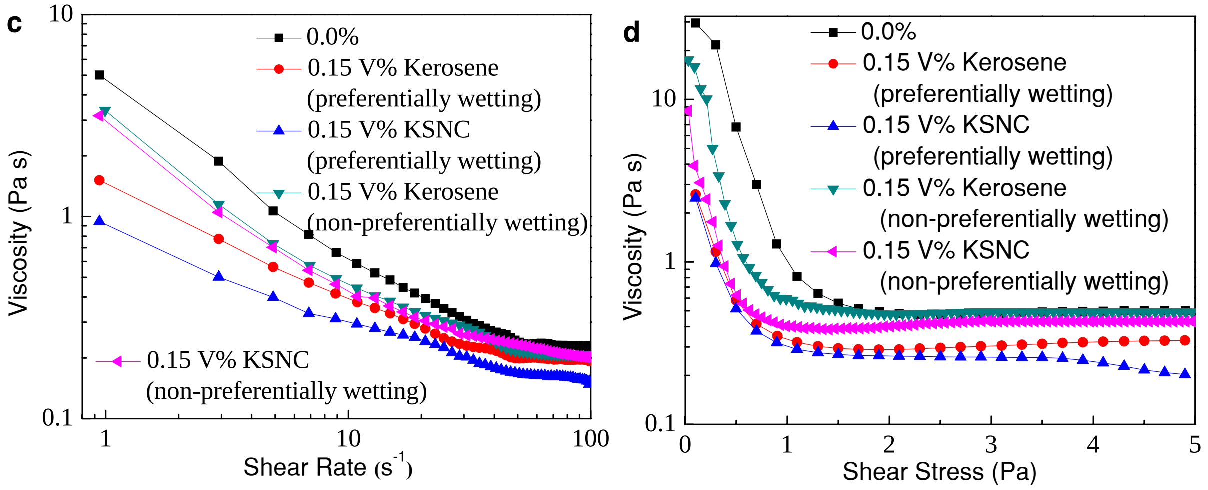

Supplementary Figure S3│ Effect of the addition of secondary fluid on the rheology of suspension prepared using PVC. Viscosity (**a**) and yield stress (**b**) at a shear rate of 100 s^{-1}, for varying volume percentages of the secondary fluid (kerosene and KSNC) that preferentially wets particles; Flow curves [(**c**) and (**d**)] of 0.15 V% (0.1 wt%) of secondary fluid (kerosene and KSNC) preferentially and non-preferentially wets particles; Error bars in (a) and (b) indicate repeatability error.

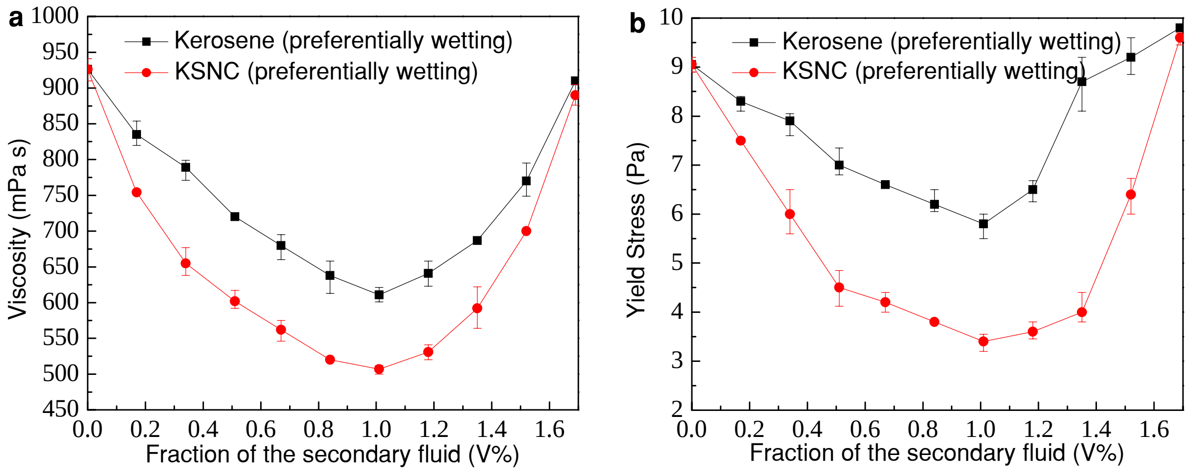

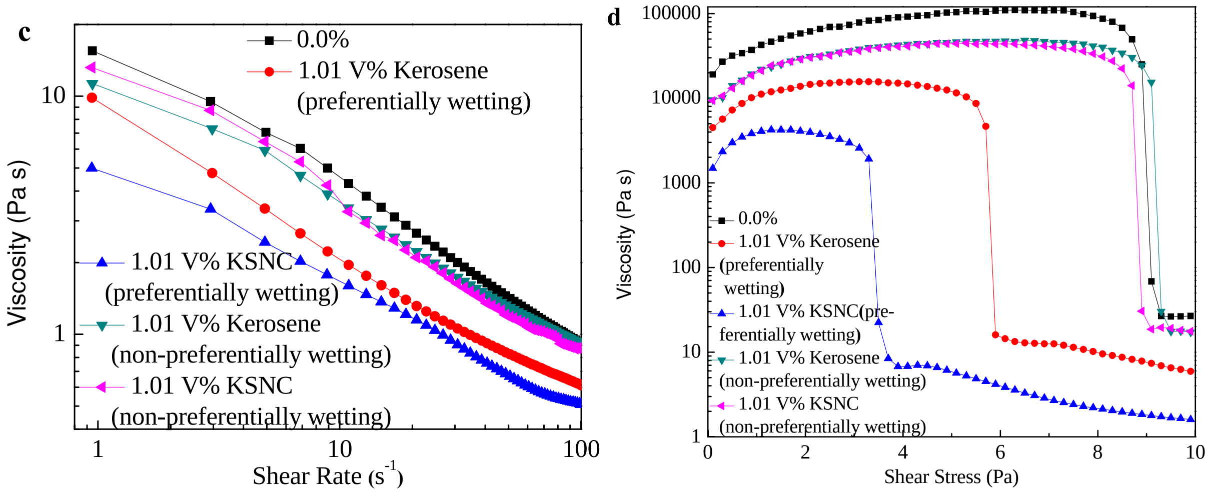

Supplementary Figure S4 | Effect of the addition of secondary fluid on the rheology of suspension prepared using Brown coal. Viscosity (**a**) and yield stress (**b**) at a shear rate of 100 s^{-1}, for varying volume percentages of the secondary fluid (kerosene and KSNC) that preferentially wets particles; Flow curves [(**c**) and (**d**)] of 1.01 V% (0.6 wt%) of secondary fluid (kerosene and KSNC) preferentially and non-preferentially wets particles; Error bars in (a) and (b) indicate repeatability error.

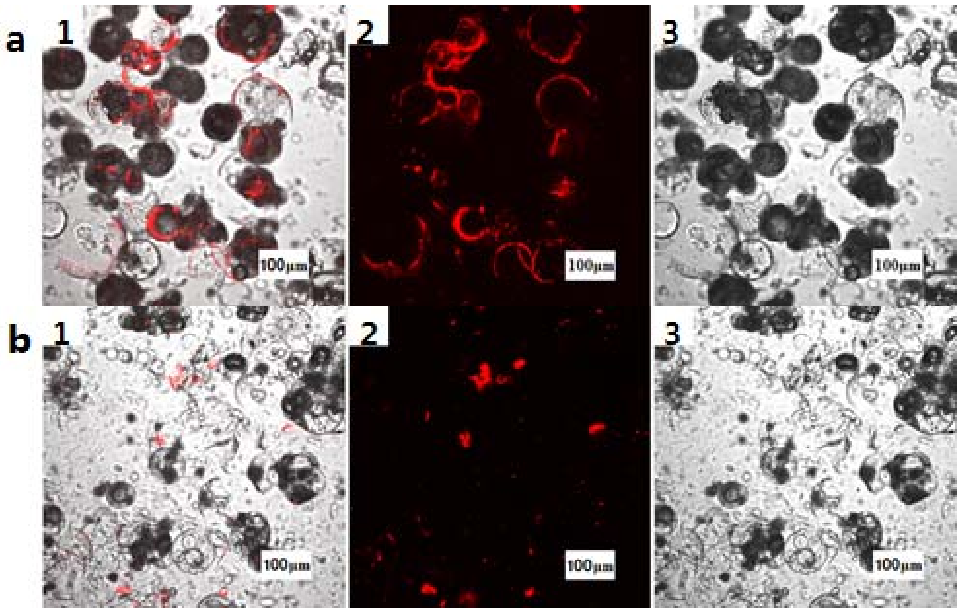

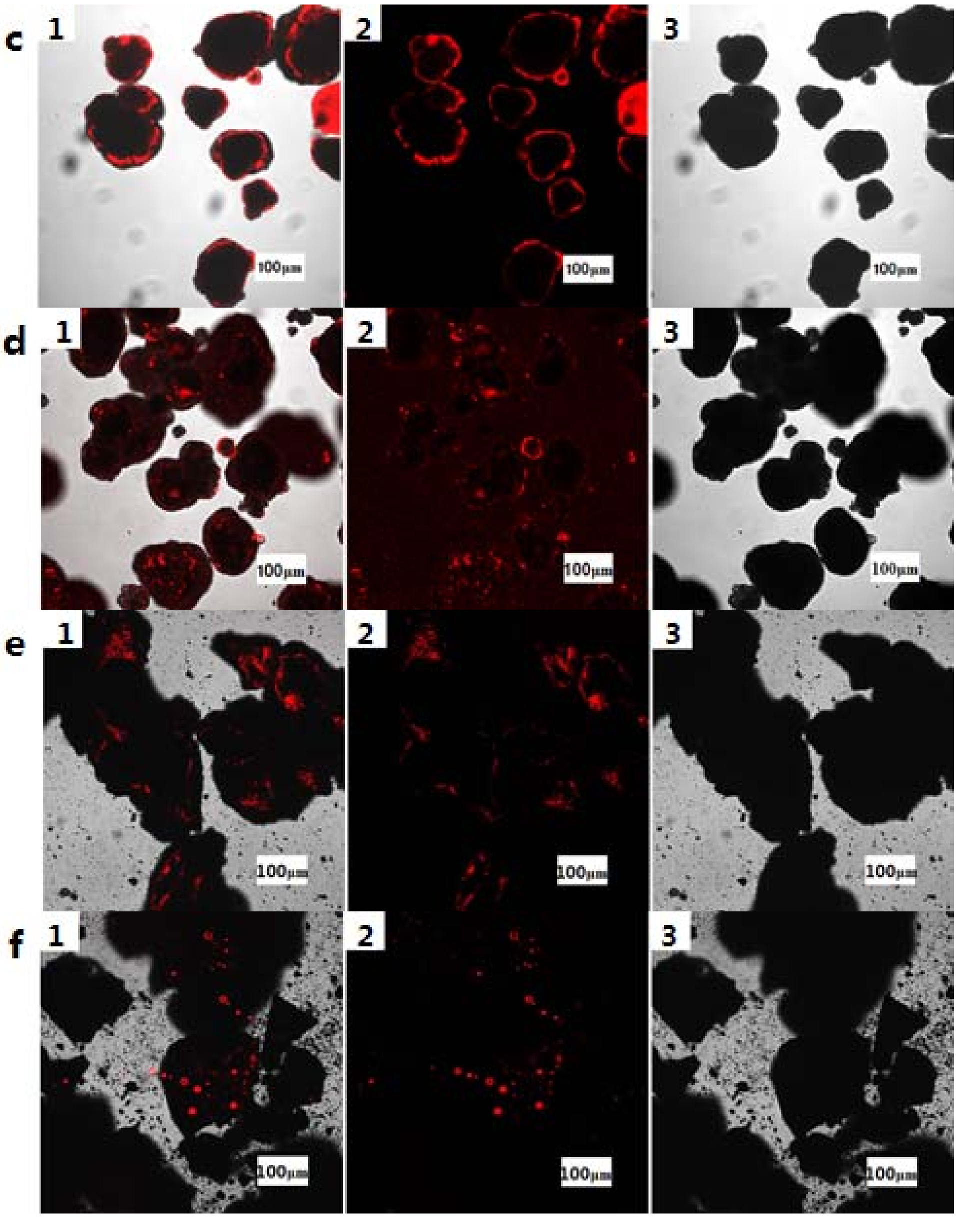

Supplementary Figure S5 | Images of forms of existence of secondary fluid in suspension. Composite images [**a**(1), **b**(1), **c**(1), **d**(1), **e**(1) and **f**(1)] mentioned in Fig.4 (see main text) are respectively composed of the hydrophobic fluorescent dye image [**a**(2), **b**(2), **c**(2), **d**(2), **e**(2) and **f**(2)] in red, used to highlight the kerosene, respectively merged onto the corresponding unfiltered, real-light images [**a**(3), **b**(3), **c**(3), **d**(3), **e**(3) and **f**(3)].

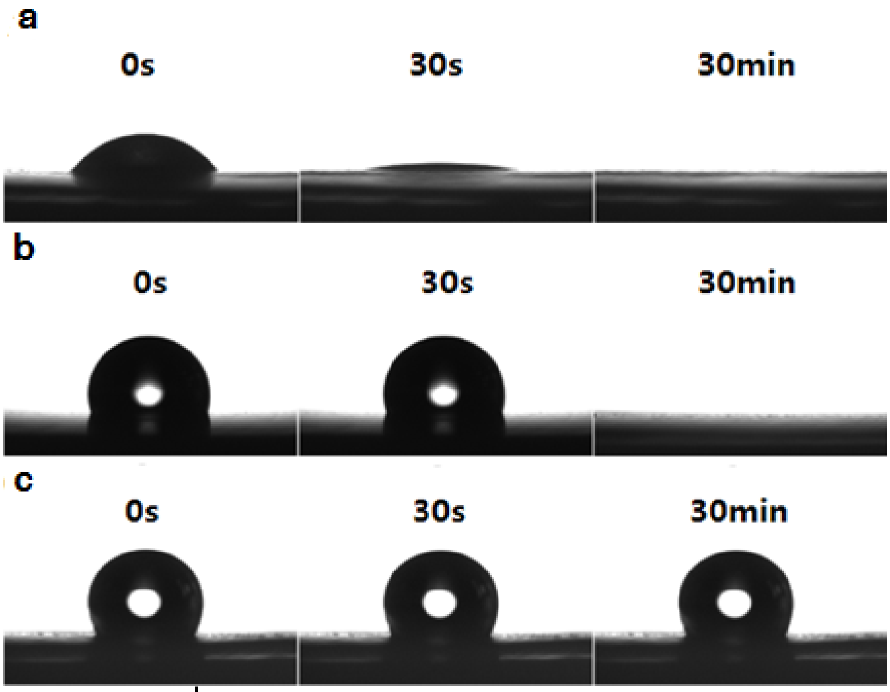

Supplementary Figure S6 | Images of a water droplet sitting on the slices of Brown coal particles in 0 s, 30 s and 30 min. a, Brown coal particles without addition of secondary fluid. **b**, Brown coal particles with 0.34 V% (0.2 wt%) of kerosene. **c**, Brown coal particles with 0.34 V% of KSNC.
